# Exploring the Relationship between Wind Patterns and Hospital Admissions Due to Respiratory Symptoms in Children

**DOI:** 10.3390/children11060717

**Published:** 2024-06-12

**Authors:** Despoina Boura, Marios Spanakis, George Markakis, George Notas, Christos Lionis, Nikolaos Tzanakis, Emmanouil Paraskakis

**Affiliations:** 1 Department of Respiratory Medicine, University Hospital of Heraklion, School of Medicine, University of Crete, 71003 Heraklion, Greece; medp1349@med.uoc.gr (D.B.); tzanakisn@uoc.gr (N.T.); 2 Department of Forensic Sciences and Toxicology, School of Medicine, University of Crete, 71003 Heraklion, Greece; marspan@ics.forth.gr; 3 Computational Bio-Medicine Laboratory, Institute of Computer Science, Foundation for Research & Technology–Hellas, 71110 Heraklion, Greece; 4 Department of Social Work, Faculty of Health Sciences, Hellenic Mediterranean University, 71004 Heraklion, Greece; gmark@hmu.gr; 5 Department of Emergency Medicine, University Hospital of Heraklion, School of Medicine, University of Crete, 71003 Heraklion, Greece; gnotas@uoc.gr; 6 Clinic of Social and Family Medicine, School of Medicine, University of Crete, 71003 Heraklion, Greece; lionis@uoc.gr; 7 Paediatric Respiratory Unit, Paediatric Department, University of Crete, 71500 Heraklion, Greece

**Keywords:** respiratory disorders, meteorological conditions, wind patterns, asthma, children, rhinitis, allergies, climatic factors

## Abstract

Respiratory disorders significantly impact adolescents’ health, often resulting in hospital admissions. Meteorological elements such as wind patterns have emerged as potential contributors to respiratory symptoms. However, it remains uncertain whether fluctuations in wind characteristics over extended periods have a tangible impact on respiratory health, particularly in regions characterized by distinct annual wind patterns. Crete is situated in the central-eastern Mediterranean Sea and frequently faces southerly winds carrying Sahara Desert sand from Africa and northerly winds from the Aegean Sea. This retrospective study analyzes long-term wind direction data and their relationship to respiratory symptoms observed in children up to 14 years old admitted at the University Hospital of Heraklion between 2002 and 2010. Symptoms such as headache, dyspnea, dry cough, dizziness, tachypnea, throat ache, and earache were predominantly reported during the presence of southern winds. Fever, productive cough, and chest pain were more frequently reported during northern winds. Cough was the most common symptom regardless of the wind pattern. Southern winds were significantly associated with higher probabilities of productive or non-productive cough, headache, dyspnea, tachypnea, dizziness, earache, and throat ache. Northern winds were related to a higher incidence of productive cough. Rhinitis, asthma, allergies, pharyngitis, and sinusitis were related to southern winds, while bronchiolitis and pneumonia were associated with northern winds. These findings underscore the critical role of local climatic factors, emphasizing their potential impact on exacerbating respiratory conditions in children. Moreover, they point out the need for further research to elucidate the underlying mechanisms and develop targeted interventions for at-risk populations.

## 1. Introduction

Asthma, bronchiolitis, and pneumonia are common respiratory disorders in children that cause dyspnea, coughing, wheezing, and chest discomfort. Respiratory disorders significantly affect the health of children worldwide, often leading to hospital admissions and impacting their overall health and well-being, as well as that of their families. For example, asthma, a major non-communicable disease (NCD), is a prevalent chronic respiratory condition affecting approximately 10–15% of the global population, primarily children, and it contributes to 1.1% of the total global estimate of “disability-adjusted life years” (DALYs) per 100,000 individuals for all causes [1]. Proper diagnosis of pediatric asthma allows for early targeted therapy, limiting the disease’s detrimental effects on patients’ quality of life [2,3].

Factors related to the development of respiratory disorders include genetic predisposition, allergies, viral infections, chemical exposure, and obesity. Furthermore, recent studies have uncovered a strong association between meteorological factors and the incidence of respiratory syndromes in children [4,5,6]. Weather-related elements, including temperature, atmospheric humidity, wind directions, and air pollution, influence the onset and progression of these pediatric disorders [7]. This association is evidenced by increased emergency department visits and admissions due to asthma exacerbations. The influence of meteorological factors, particularly temperature, on asthma prevalence and hospitalizations has been thoroughly examined [8]. Previous studies have associated cold and hot exposures with increased asthma risk [9,10,11,12]. Epidemiological data suggest a strong association between cold weather and increased asthma-related hospital admissions, impacting both adults and infants [13,14,15,16,17]. However, the precise correlation between ambient temperature and asthma-related hospitalizations remains inadequately understood [18]. Conversely, the impact of warmer temperatures on asthma prevalence has been validated in only a limited number of recent studies and in regions with different meteorological characteristics [19,20,21]. Generally, temperature fluctuations and humidity levels potentially cause respiratory epithelium constriction and heightened sensitivity, worsening respiratory symptoms, especially for individuals with pre-existing conditions [22,23,24]. Moreover, young children are highly susceptible to acute lower respiratory infections (ALRIs), with climate conditions contributing significantly to seasonal fluctuations in viral ALRIs [25,26,27,28].

Considering meteorological conditions and their significant regional and seasonal variability, it is essential to utilize area-specific meteorological data to uncover potential associations with the incidence of respiratory disorders in vulnerable populations, such as children at risk from asthma exacerbations [29,30,31]. Understanding the impact of meteorological factors in a world with accelerating climatic change is vital for the design of effective respiratory health interventions [32]. Moreover, studies suggest that, to adapt to region-specific weather dynamics, localized analysis is crucial for tailoring effective interventions [32,33,34].

**Figure 1:**
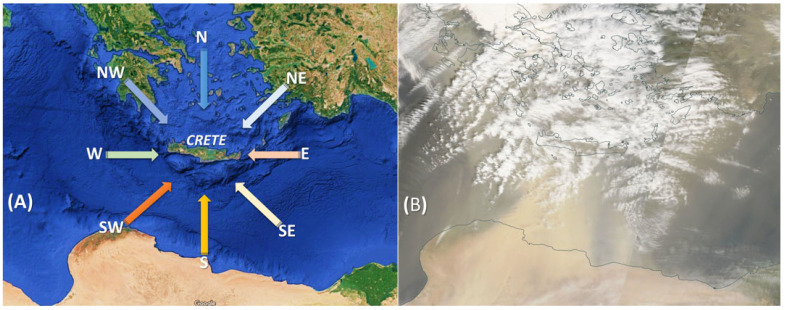
(**A**) Location of the island and wind directions (source Google Maps). (**B**) Desert dust arrived from Africa on 22 March 2018 during a SW-S windstorm [42].

Crete, located in the central-east Mediterranean Sea, has a typical Mediterranean climate, with mean temperatures ranging from approximately 14 °C in winter to 32 °C in summer. Northern winds in Crete usually bring colder temperatures and humidity, which are associated factors that increase the overall incidence of respiratory infections. On the other hand, proximity to Africa exposes Crete to southerly winds, which are generally warmer but also carry sand from the Sahara and Arabian deserts (Figure 1) [30,35,36,37,38]. This distinctive environmental setting prompts an investigation into whether specific wind characteristics are linked to childhood respiratory disorders [39,40,41]. The aim of this work is to thoroughly explore possible associations between distinct wind patterns and respiratory disorders. The study focuses on infants and adolescents, crucial groups due to the increased incidence of respiratory disorders, particularly asthma, during this developmental stage. Evaluating a decade of data from emergency department admissions to the University Hospital of Heraklion, the dataset is correlated with weather information from the same period to identify potential links or associations between wind patterns, symptoms, examinations, and diagnoses.

## 2. Materials and Methods

### 2.1. Study Design and Target Population

The retrospective observational study included anonymized data from medical records of infants and adolescents (0–14 years) admitted from 2002 to 2010 to the emergency department of the University Hospital of Heraklion, Crete, with various symptoms. Age, gender, date, symptoms, and examination results were extracted and analyzed. Among the diagnoses examined were asthma exacerbations, respiratory infection, bronchiolitis, pneumonia, rhinitis, sinusitis, pharyngitis, otitis, allergy, and croup.

### 2.2. Wind Conditions

The atmospheric conditions were documented using the Hellenic National Meteorological Service (HNMS) information for the same period (2002–2010). We obtained daily data on the prevailing wind direction, average and maximum wind speeds, temperature, and humidity levels. Wind direction is expressed as north (N), northeast (NE), east (E), southeast (SE), south (S), southwest (SW), west (W), and northwest (NW). Wind speed (WS) is expressed in knots, temperature (T) in Celsius (°C), and humidity (H) as percentage (relative humidity).

### 2.3. Statistical Analysis

The data above were input individually into the base of the IBM-SPSS 22.0 software. Continuous variables were expressed as mean values ± standard deviation (SD). To investigate the relationship between the wind direction and the existence of various respiratory conditions, a series of multivariate binary logistic regression models were used for the prediction of symptoms, where symptoms are binary variables. For each symptom [cough (0 or 1), dry cough (0 or 1), productive cough (0 or 1), tachypnea (0 or 1), dyspnea (0 or 1)], five different binary regression models have been constructed with different dependent variables. The model’s predictions include the recorded wind direction (1 = north, 2 = northeast, 3 = east, 4 = southeast, 5 = south, 6 = southwest, 7 = west, 8 = northwest, 9 = no wind). Each wind direction was compared to no wind conditions (<1 knot), serving as the reference point for all winds. Adjusted odds ratios (AORs) as to wind speed (WS), maximum wind speed (WSmax), humidity (H), and temperature (T), along with the associated 95% confidence intervals (CIs), were calculated with *p*-values < 5% set as the criterion for significance.

### 2.4. Bioethics

The study was approved by the University Hospital of Heraklion Ethics Committee (no. 38246/10-11-2023). All data extracted from the medical records were anonymous and contained non-identifiable information. As a retrospective study on anonymized data dating over a decade, informed consent was exempted.

## 3. Results

### 3.1. Hospital Admissions 2002–2010

**Figure 2:**
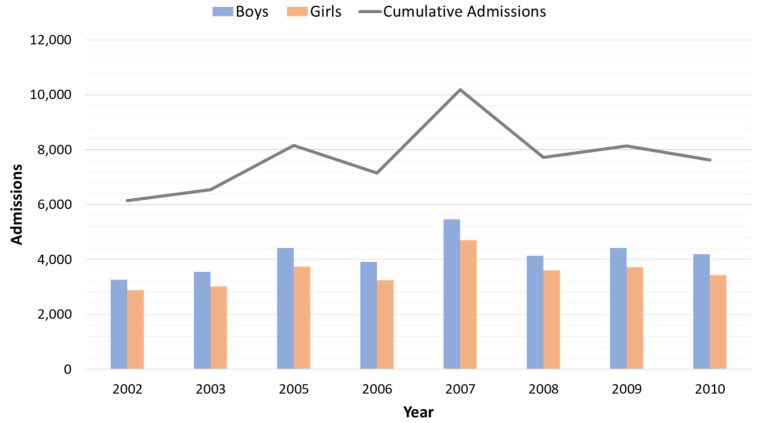
Annual hospitalizations in the pediatric department of the University Hospital of Heraklion for respiratory conditions.

The study examined 62,477 medical records (35,607 boys and 23,469 girls) from hospital admissions in the emergency department of children with respiratory conditions or related symptoms between 2002 and 2010, except for 2004, due to data retrieval issues for this year. Among these records, 797 had missing values, leaving 61,680 unique records that met the inclusion criteria. On average (±SD), there were 7710 admissions yearly (±1153). The lowest number of admissions per year was recorded in 2002 (6141) and the highest in 2007 (10,184). Boys were more frequently admitted, with an average of 4168 (±624) admissions per year versus 3542 (±532) admissions for girls. The lowest number of admissions for both boys and girls were in 2002, with 3260 and 2881, respectively. The higher frequency of admissions for boys was statistically significant (*p* < 0.05). Figure 2 demonstrates the hospital visit admissions per year for each gender group. Overall, there was a characteristic peak in admissions during 2007, whereas in later years, the admissions remained relatively higher than they were prior to 2007.

### 3.2. Wind Characteristics 2002–2010

**Figure 3:**
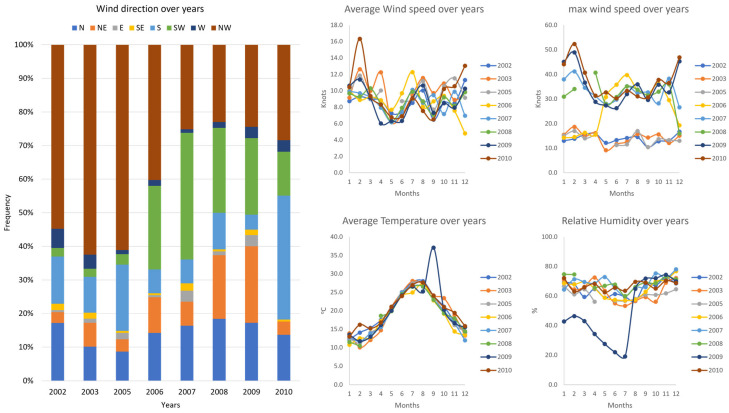
Wind characteristics as recorded for the period 2002–2010 in Crete. All data are expressed as mean values for each month of the year.

Information obtained from the HNMS for the same period (2002–2010) regarding wind characteristics is depicted in Figure 3. The winds were mainly from the NW direction throughout the years, but there was a significant increase in S and SW frequency from 2006 to 2010. Wind speed followed a similar trend, ranging from 4 to 16 knots. As for the max wind speed, three distinct periods can be described: the first refers to the years 2002 to 2005 with relatively low max speeds, the second refers to the year 2006 with both low and high winds, and finally, the third refers to the years 2007 to 2010, where max wind speeds display higher values. Crete’s mean temperature over the years seems to follow the typical Mediterranean seasonal fluctuation, with a minor exception in September 2009, where a higher mean temperature was recorded. The relative humidity again shows similar trends throughout the years, except for the first semester of 2009, which had relatively drier winds.

### 3.3. Symptoms, Examinations, and Diagnoses for the Period 2002–2010

**Figure 4:**
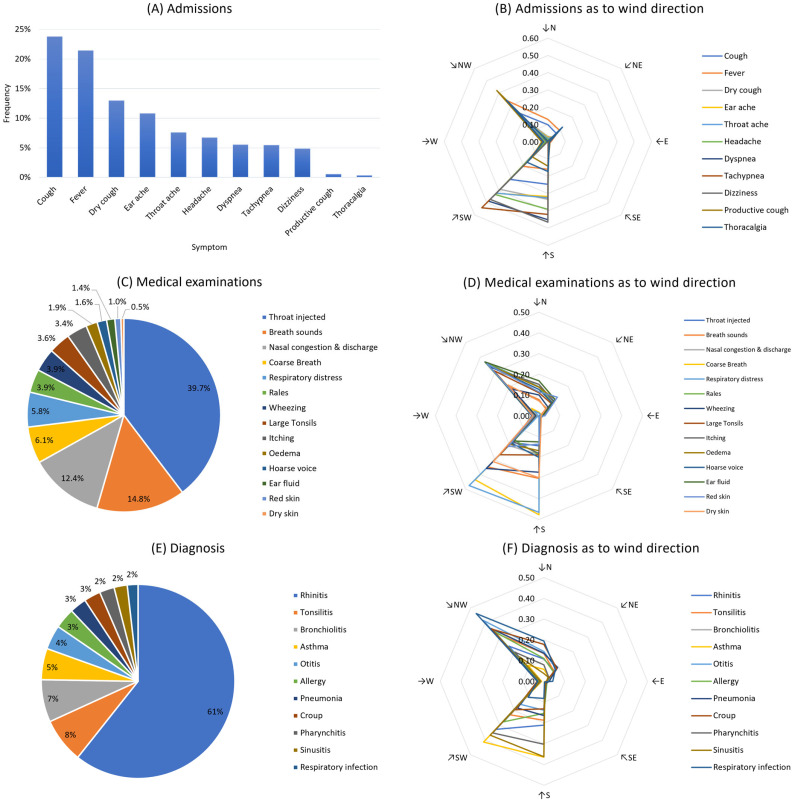
The dataset contains reported symptoms, medical examinations, diagnoses, and their frequencies throughout the years as to the prevailing wind direction. (**A**) Frequencies of reported symptoms upon admission; (**B**) Admissions as to wind direction; (**C**) Physical examination findings; (**D**) Examination findings as to wind direction; (**E**) Diagnoses and (**F**) Diagnoses as to wind direction; (**B**,**D**,**F**) radar plots vertical axes referring to frequencies.

Analysis of medical records from 2002 to 2010 revealed that the most frequently reported symptoms upon admission, linked to cases of respiratory distress, included cough, fever, headache, dry cough, earache, throat ache, headache, dyspnea, tachypnea, dizziness, productive cough, and chest pain (Figure 4A). Considering the accumulated frequencies of reported symptoms relative to respiratory conditions, they can be grouped into two groups regarding prevailing winds (Figure 4B). The first category is for prevailing southern winds (S and SW). Symptoms such as cough, headache, dyspnea, dry cough, dizziness, tachypnea, throat ache, and earache were mostly reported when S and SW winds were present. The most frequently reported symptoms when northern winds were present (NW, N, and NE) were fever, cough, productive cough, and chest pain. Cough was a common symptom reported during both southern and northern winds.

The analysis of symptoms from the physical examination reveals that the most often reported findings were throat injection, abnormal lung sounds, nasal congestion and discharge, coarse breath, and respiratory distress (Figure 4C). As for the wind directions (Figure 4D), two distinct clusters can be observed, one referring to southern winds and one to northern winds. Southern winds (S, SW) were more commonly correlated with abnormal breath sounds, coarse breath, wheezing, and certain ear, nose, and throat symptoms. In contrast, northern winds (N, NW) were associated with higher frequencies of throat injected, rales, itching, edema, ear fluid, and red skin. Hoarse voice and respiratory distress appeared in both wind directions, suggesting comparable associations.

Following symptom analysis and diagnostic process evaluation, 31,672 reports, or 51.3% of the medical records examined, were related to respiratory syndromes and were grouped into 11 distinct diagnoses (Figure 4E). The most frequent diagnosis was rhinitis (allergic and non-allergic), reported in 61.8% of the subjects, followed by tonsillitis, bronchiolitis, asthma, otitis, and allergy in rates between 3 and 8%. The representation of the reported wind direction is presented in Figure 4F. Again, two clusters of diagnoses can be sorted considering prevailing wind directions. Southern winds (S, SW) were associated with higher frequencies of asthma, allergy, pharyngitis, and sinusitis diagnoses. In comparison, northern winds showed higher frequencies of bronchiolitis, tonsillitis, otitis, pneumonia, croup, and respiratory infection diagnoses.

### 3.4. Associations of Symptoms, Physical Examination Findings, and Diagnoses with Wind Direction

Depending on the presence or absence of wind, logistic regression models were used to forecast symptoms, physical examinations, and diagnoses (Table 1, Table 2 and Table 3). The analysis of symptoms reveals that southern winds (S and SW) were positively associated with significantly higher odds (*p* < 0.05) for cough (e.g., S: OR = 3.536); dry cough (e.g., S: OR = 3.794, and SW: OR = 2.996); headache (e.g., S: OR = 2.899); dyspnea (e.g., S: 3.067); tachypnea (e.g., S: 2.626); dizziness (e.g., S: 4.340 and SW: OR = 2.952); earache (e.g., S: OR = 2.133) and chest pain (e.g., S: OR = 2.081). For all other cases, the adjusted odds ratio to other confounders (WS, H, T) showed an inverse correlation or no contribution to the prevalence of symptoms. Northern winds were associated with significantly higher odds (*p* < 0.05) for productive cough (e.g., N: OR = 6.032, NE: OR = 5.804, and NW: OR = 6.101). Productive cough was also associated with high odds with W winds (OR = 7.085). The rest of the wind directions seemed to have no association with all other symptoms or were inversely correlated with their appearance.

The analysis of physical diagnosis as to the prevailing wind conditions suggests that winds from the S and SW directions show notable associations with findings such as abnormal breath sounds (e.g., S: OR = 2.530), nasal congestion or discharge (e.g., S: OR = 1.736), dyspnea (e.g., S: OR = 3.094, and SW: OR = 1.950), respiratory distress (e.g., S: OR = 3.318, and SW: OR = 2.386), rales (e.g., S: OR = 2.898), wheezing (e.g., SW: OR = 1.861) and enlarged tonsils (e.g., SW: OR = 1.721). Northern winds and those mainly of the NW direction were primarily associated with significantly higher odds for rales (e.g., NW: OR 2.116) and, to a lesser extent, with a hoarse voice (e.g., NW: OR 1.737). As for other wind directions, western winds showed a significant association (*p* < 0.05) for rales (OR: 2.083) and hoarse voice (OR: 2.488). Regarding all other findings, wind directions appeared to have no or a reverse association with them.

Regarding final diagnoses, wind directions significantly correlated with certain respiratory conditions. Rhinitis, the most often diagnosed condition, showed significant associations with S (OR = 2.189) and SW (OR = 2.993) winds. The presence of southern winds was also associated with the diagnosis of asthma (SW: OR = 1.800), allergies (SW: OR = 2.035), pharyngitis (SE: OR = 4.270 and SW: OR = 3.785), and sinusitis (SW: OR = 2.930). Northern winds showed a significant positive association with bronchiolitis (NW: OR = 3.707). Other diagnoses (i.e., otitis, croup, and respiratory infection), although often reported, did not reveal any positive associations with a specific wind pattern.

## 4. Discussion

The findings of the current analysis show the potential impact of wind patterns on the respiratory health of adolescents in regions like Crete, characterized by distinct wind conditions. In this study, children’s admissions in a tertiary hospital pediatric emergency department can be categorized into the following two clusters based on wind direction in regard to symptoms, examinations, and final diagnoses: a southern winds cluster and a northern winds cluster. Western winds exhibited a significant association, only featuring a productive cough and hoarse voice. An important observation is the correlation between specific wind directions, notably S and SW winds, and a range of respiratory symptoms and diagnoses.

The observed association between specific wind directions and respiratory distress symptoms such as cough, dry cough, tachypnea, and dyspnea, along with physical examination findings, implies a possible influence of these specific winds on the respiratory health of adolescents in Crete. Additionally, the strong correlation identified between these winds and diagnoses, including rhinitis, sinusitis, and pharyngitis, and especially the substantial odds ratio for asthma, underscores the potential impact of wind patterns on the exacerbation and prevalence of respiratory conditions in this region. It should also be noted that there is no display of differences in asthma prevalence between young children from rural and urban areas [43]. The prevalent diagnosis of rhinitis, either seasonal, perennial, or occupational, should be considered further, since rhinitis has been described in several studies as a predecessor of asthma development [44,45]. Moreover, several studies suggest that some types of rhinitis and sinusitis (i.e., persistent allergic rhinitis and chronic rhinosinusitis) are often present in asthmatic patients [46,47]. As for pharyngitis, it is a risk factor for asthma exacerbations due to infectious agents [48]. Similarly, the frequent diagnosis of tonsilitis, as well as potential tonsillar hypertrophy, are possible risk factors for asthma since they are often associated with asthma-related symptoms [49]. Interestingly, all of these conditions, along with their accompanying symptoms, were positively related to the appearance of southern winds, whereas other wind directions appear to have a reverse or no contribution.

The appearance of S and SW in Crete is often accompanied by sand dust from the Sahara Desert (Figure 1). Studies conducted in similar climatic settings have reported increased hospital admissions and respiratory disorders linked to elevated particulate matter levels resulting from desert dust storms [50]. Previous studies have suggested significant associations between desert dust exposure and asthma exacerbation, revealing an increased risk of hospitalization and a decline in lung function in children and adults [51,52,53]. Additional studies further explored this relationship, finding that pollen can augment the impact of desert dust on asthma symptoms and noting a variable influence of desert dust on lower respiratory tract symptoms in adult asthma patients [54,55]. These findings suggest that desert dust can exacerbate asthma, particularly when combined with other environmental factors. Seasonal meteorological factors such as humidity, barometric pressure, temperature, and wind speed have been linked to asthma exacerbations in children, each with varying degrees of significance [8,56], which was replicated in our study. However, our study introduces another parameter associated with asthma and related symptoms and conditions, namely wind direction. This parameter must also be considered, considering its impact on air quality based on the origin of the wind, regardless of its proximity to the area [57]. Suspended dust particles in the atmosphere for prolonged periods can instigate respiratory disorders and allergy outbreaks in regions far from their origins, depending on the climatic conditions. Therefore, meteorological conditions facilitating the transport of desert dust can lead to local environmental alterations, prolonging the presence of factors that trigger respiratory syndromes for extended periods and increase hospitalizations [58]. Research findings and air quality monitoring networks strongly link Saharan dust intrusions in Southern Europe to escalating particulate matter (PM), anthropogenic pollution conveyance or other pollutant concentrations, and potential microorganism transfer, citing them as contributing factors to worsening multiple pathologies [57,59,60]. As for children, earlier studies on respiratory disorders have associated heightened PM levels during Sahara dust episodes with increased asthma exacerbations [61]. In our approach, this was made further evident by associated symptoms such as cough, dry cough, tachypnea, and dyspnea, coupled with physical examination findings. These clinical signs, commonly observed and strongly correlated with asthma or asthma-related conditions, were also significantly associated with S and SW winds. Such winds, at least on the island of Crete, increase desert dust transport and subsequently diminish air quality. This observation is also supported by the peak in admission rates after 2006, which follows the increased incidence of SW winds over the same time (Figure 2 and Figure 3).

The second cluster of symptoms, physical examination findings, and diagnoses in this study are related to northern winds. Interestingly, these symptoms and diagnoses mostly correlate with specific weather conditions due to wind direction [41]. In Crete, northern winds often bring colder temperatures and higher humidity levels. These weather changes can significantly affect respiratory health, potentially leading to the development or worsening of respiratory disorders. The cold air associated with northern winds can cause airway constriction, triggering symptoms like productive coughing and exacerbating existing respiratory conditions [37]. The increased humidity brought by these winds creates a conducive environment for respiratory pathogens to thrive, increasing the risk of infections such as bronchitis and pneumonia [62]. Furthermore, the scattering of allergens and air pollutants by northern winds could have a positive impact, reducing the incidence of symptoms associated with asthma-related conditions, as the data show (Table 1, Table 2 and Table 3) [63].

A strength of this study is the comprehensive nature of the data analysis involving a large sample size drawn from 61.680 unique medical records. This extensive dataset covering an extended period allows for robust observations and statistical associations regarding the link between meteorological conditions, such as wind directions and respiratory health indicators for young children. Moreover, adjusting for confounders such as wind speed, humidity, and temperature allows for a more accurate estimation of the association between the predictor variable (wind direction) and the outcome variable (symptoms, physical findings, and diagnoses) by controlling for potential sources of bias or distortion in the data. However, considering the thorough interpretation of the study’s findings, several limitations should be mentioned. While this study establishes a strong link between wind directions and respiratory symptoms in adolescents, it is essential to acknowledge the complexity of factors contributing to respiratory disorders. Other environmental factors, such as air pollution, PM2.5 and PM10 concentrations, pollen count, and other seasonal allergens, are likely carried by these winds and could contribute to wind patterns, potentially influencing respiratory health outcomes. These variables might act independently or synergistically, necessitating comprehensive multivariable analyses to understand their collective impact better. The retrospective observational nature of the study, reliant solely on medical records, might introduce biases and overlook pertinent environmental or individual variables contributing to respiratory conditions. Moreover, respiratory distress symptoms may have been initiated on a day different from the admission day. Thus, while the study identified a correlation between wind patterns and respiratory symptoms, establishing a causal relationship requires additional investigations. For example, the impact of wind speed on the development of respiratory disorders should also be considered along with wind direction [64,65]. Longitudinal studies incorporating detailed environmental data alongside individual health records could offer insights into the mechanisms through which specific wind patterns might affect respiratory health. Therefore, the study underscores the need for further prospective research integrating broader environmental data and individual health profiles to elucidate causal mechanisms better and comprehensively address potential confounders [66,67].

Our findings emphasize the importance of considering local climatic factors in understanding and managing respiratory diseases, especially in regions like Crete, which feature distinct annual wind patterns. Such insights could inform public health interventions, healthcare planning, and patient education to mitigate the impact of environmental factors on respiratory health. Collaborative efforts involving meteorologists, healthcare professionals, and policymakers might be crucial in developing strategies to minimize the adverse effects of specific wind patterns on adolescent respiratory health.

## 5. Conclusions

This retrospective observational study analyzed long-term wind patterns in Crete and their link to adolescent respiratory conditions/dysfunction. It found a strong association between S and SW winds and various respiratory symptoms, like coughing, along with specific diagnoses such as rhinitis, sinusitis, and, most importantly, asthma. Understanding the influence of local climatic factors, especially wind directions, is crucial for managing adolescent respiratory conditions. Although further research is needed to include elements like air pollution and humidity to establish causal relationships, these findings can guide targeted healthcare planning and develop effective strategies to minimize the adverse effects of specific wind patterns on respiratory health in such regions.

## Figures and Tables

**Table 1:** Logistic regression models predict the symptoms reported upon admission, based on occurring wind direction. ORs are calculated against no wind conditions adjusted by WS, WSmax, H and T. The (*) indicates significance at *p* < 0.05. Bold font highlights ORs > 1 with corresponding *p*-values < 0.05.

	Wind Direction							
	N	NE	E	SE	S	SW	W	NW
Symptoms	OR 95%CI	OR 95%CI	OR 95%CI	OR 95%CI	OR 95%CI	OR 95%CI	OR 95%CI	OR 95%CI
**Cough**	0.424 * 0.361–0.499	0.481 * 0.408–0.567	0.429 * 0.351–0.526	0.519 * 0.49–0.643	3.536 * **3.003–4.165**	2.729 * **2.324–3.206**	0.404 * 0.335–0.487	0.443 * 0.378–0.520
**Fever**	0.859 0.731–1.008	0.816 0.693–0.960	0.732 0.600–0.893	0.980 0.795–1.207	1.153 0.981–1.355	1.030 0.878–1.209	0.904 0.752–1.087	0.852 0.727–0.999
**Dry cough**	0.280 * 0.232–0.339	0.269 * 0.221–0.328	0.154 * 0.110–0.214	0.191 * 0.136–0.269	3.794 * **3.169–4.541**	2.996 * **2.506–3.581**	0.210 * 0.160–0.276	0.349 * 0.291–0.418
**Earache**	0.206 * 0.170–0.249	0.223 * 0.183–0.271	0.227 * 0.17–0.300	0.236 * 0.174–0.321	2.133 * **1.784–2.551**	2.105 * **1.763–2.513**	0.239 * 0.188–0.305	0.215 * 0.179–0.258
**Throat ache**	0.247 * 0.198–0.308	0.235 * 0.186–0.297	0.237 * 0.169–0.333	0.200 * 0.135–0.298	2.081 * **1.693–2.559**	2.018 * **1.643–2.477**	0.266 * 0.199–0.355	0.235 * 0.190–0.290
**Headache**	0.166 * 0.129–0.214	0.182 * 0.140–0.238	0.183 * 0.122–0.275	0.274 * 0.184–0.407	2.899 * **2.316–3.629**	2.195 * **1.755–2.745**	0.262 * 0.191–0.359	0.177 * 0.140–0.224
**Dyspnea**	0.048 * 0.034–0.066	0.052 * 0.036–0.076	0.012 * 0.003–0.050	0.115 * 0.065–0.205	3.067 * **2.424–3.880**	2.240 * **1.772–2.832**	0.074 * 0.045–0.122	0.060 * 0.046–0.079
**Tachypnea**	0.032 * 0.022–0.047	0.037* 0.024–0.056	0.012 * 0.003–0.049	0.032 * 0.011–0.086	2.626* **2.075–3.324**	2.450* **1.938–3.097**	0.048 * 0.027–0.086	0.032 * 0.024–0.043
**Dizziness**	0.069 * 0.047–0.101	0.072 * 0.047–0.110	0.039 * 0.014–0.107	0.201 * 0.112–0.359	4.340 * **3.262–5.774**	2.952 * **2.220–3.926**	0.79 * 0.044–0.144	0.088 * 0.064–0.120
**Productive cough**	6.032 * **0.836–43.502**	5.804 * **0.800–42.109**	1.780 0.185–17.160	6.519 0.822–51.706	5.551 0.767–40.143	3.594 0.496–26.040	7.085 * **0.947–53.012**	6.101 * **0.849–43.846**
**Chest pain**	1.796 0.433–7.444	2.216 0.533–9.209	1.205 0.220–6.611	1.534 0.279–8.442	2.265 0.547–9.374	1.668 0.403–6.906	1.711 0.365–8.016	1.587 0.386–6.521

**Table 2:** Logistic regression models associate physical examination as documented by the treating physician and wind direction. ORs are calculated against no wind conditions adjusted by WS, WSmax, H and T. The (*) indicates significance at *p* < 0.05. Bold font highlights ORs > 1 with corresponding *p*-values < 0.05.

	Wind Direction							
	N	NE	E	SE	S	SW	W	NW
Physical Examination	OR 95%CI	OR 95%CI	OR 95%CI	OR 95%CI	OR 95%CI	OR 95%CI	OR 95%CI	OR 95%CI
**Throat injected**	0.823 0.701–0.965	0.955 0.813–1.122	1.158 0.954–1.406	1.037 0.844–1.275	0.986 0.840–1.157	0.883 0.753–1.035	0.948 0.790–1.139	0.904 0.772–1.058
**Breath sounds** **(wet, echo, dry)**	0.560 0.450–0.696	0.488 0.389–0.611	0.307 * 0.228–0.433	0.500 0.359–0.696	2.530 * **2.054–3.117**	2.171 * **1.763–2.673**	0.580 0.444–0.776	0.572 0.464–0.706
**Nasal Congestion/Discharge**	1.293 1.001–1.612	1.123 0.872–1.445	1.175 0.870–1.574	1.184 0.863–1.623	1.736 * **1.356–2.221**	1.407 * **1.103–1.800**	1.565 1.192–2.057	1.035 0.811–1.321
**Coarse breath**	0.083 0.059–0.121	0.089 0.059–0.134	0.022 0.005–0.089	0.202 0.110–0.369	3.094 * **2.301–4.160**	1.950 * **1.450–2.622**	0.108 0.060–0.191	0.093 0.067–0.129
**Respiratory distress**	0.069 * 0.045–0.105	0.047 * 0.046–0.117	0.025 * 0.006–0.103	0.067 * 0.024–0.186	3.318 * **2.424–4.424**	2.386 * **1.744–3.263**	0.077 * 0.038–0.153	0.066 * 1.004–1.018
**Rales**	1.883 * **1.093–3.243**	1.958 * **1.132–3.387**	1.141 0.589–2.213	2.184 1.163–4.102	2.898 * **1.686–4.980**	1.791 * **1.041–3.080**	2.083 * **1.162–3.732**	2.116 * **1.234–3.629**
**Wheezing**	0.607 * 0.410–0.898	0.559 * 0.373–0.838	0.548 * 0.321–0.936	0.274 * 0.133–0.566	1.870 * **1.280–2.733**	1.861 * **1.277–2.713**	0.416 * 0.249–0.695	0.451 * 0.307–0.662
**Large Tonsils**	0.857 0.553–1.329	0.916 0.588–1.426	0.701 0.396–1.240	0.682 0.364–1.277	1.627 * **1.056–2.505**	1.721 * **1.122–2.641**	0.965 0.582–1.598	0.991 0.645–1.523
**Itching**	1.410 0.878–2.264	1.211 0.750–1.956	1.248 0.707–2.203	1.135 0.611–2.107	1.878 * **1.171–3.012**	1.360 0.848–2.181	1.848 1.096–3.115	1.251 0.782–2.001
**Oedema**	1.406 0.711–2.779	1.583 0.798–3.140	1.043 0.442–2.461	0.605 0.214–1.716	1.915 0.970–3.780	1.752 0.890–3.452	1.486 0.684–3.229	1.381 0.703–2.710
**Hoarse Voice**	1.617 0.707–3.699	1.631 0.708–3.757	1.555 0.594–4.072	1.563 0.574–4.259	2.694 * **1.184–6.131**	1.936 0.851–4.407	2.488 * **1.031–6.003**	1.737 * **0.765–3.942**
**Ear fluid**	2.083 0.847–5.124	1.996 0.807–4.938	1.749 0.626–4.887	1.790 0.617–5.194	1.653 0.667–4.094	1.767 0.718–4.350	1.905 0.716–5.063	1.798 0.734–4.406
**Red skin**	1.485 0.537–4.104	2.306 0.839–6.339	1.046 0.293–3.727	0.639 0.154–3.115	1.873 0.679–5.167	2.162 0.791–5.913	1.939 0.635–5.918	1.884 0.691–5.140
**Dry skin**	0.561 0.166–1.895	0.569 0.64–1.977	0.651 0.131–3.241	1.422 0.337–6.003	2.561 0.800–8.196	2.059 0.644–6.580	1.336 0.356–5.010	0.657 0.203–2.131

**Table 3:** Logistic regression models associate medical diagnoses as documented by the treating physician and wind direction. ORs are calculated against no wind conditions adjusted by WS, WSmax, H and T. The (*) indicates significance at *p* < 0.05. Bold font highlights ORs > 1 with corresponding *p*-values < 0.05.

	Wind Direction							
	N	NE	E	SE	S	SW	W	NW
Diagnoses	OR 95%CI	OR 95%CI	OR 95%CI	OR 95%CI	OR 95%CI	OR 95%CI	OR 95%CI	OR 95%CI
**Rhinitis**	0.667 * 0.562–0.791	0.689 * 0.579–0.820	0.634 * 0.511–0.786	0.559 * 0.441–0.708	2.189 * **1.848–2.594**	2.993 * **2.530–3.541**	0.638 * 0.523–0.778	0.635 * 0.537–0.751
**Tonsillitis**	0.945 0.638–1.399	0.676 0.451–1.014	0.740 0.441–1.240	1.097 0.660–1.823	1.486 * **1.008–2.192**	1.383 0.940–2.034	1.081 0.684–1.706	0.970 0.659–1.426
**Bronchiolitis**	1.365 * **0.833–2.241**	1.37 * **0.830–2.261**	0.683 0.353–1.325	1.393 0.764–2.539	1.722 1.052–2.819	1.470 0.899–2.401	0.420 0.213–0.828	3.707 * **2.208–6.028**
**Asthma**	0.250 * 0.162–0.385	0.199 * 0.124–0.319	0.377 * 0.205–0.695	0.115 * 0.040–0.328	1.800 * **1.215–2.666**	1.530 * **1.035–2.264**	0.245 * 0.133–0.453	0.185 * 0.122–0.281
**Otitis**	0.29 0.543–1.592	0.863 0.498–1.494	0.887 0.453–1.735	0.96 0.496–1.996	1.006 0.587–1.726	0.841 0.492–1.439	1.138 0.624–2.076	1.136 0.669–1.929
**Allergy**	0.900 0.501–1.616	0.712 0.390–1.300	1.106 0.531–2.303	1.829 0.904–3.702	1.452 * **0.814–2.590**	2.035 * **1.151–3.595**	1.294 0.644–2.602	1.146 0.647–2.028
**Pneumonia**	3.868 * **0.530–28.258**	4.403 * **0.600–32.287**	0.619 0.039–9.922	5.828 0.712–47.725	3.326 0.452–24.456	2.498 0.340–18.349	4.883 0.632–37.728	2.546 0.349–18.579
**Croup**	1.157 0.620–2.160	0.896 0.473–1.700	0.535 0.220–1.300	0.792 0.333–1.883	0.952 0.506–1.790	1.022 0.548–1.904	1.076 0.528–2.192	0.966 0.520–1.796
**Pharyngitis**	0.960 0.412–2.238	0.509 0.207–1.248	1.363 0.500–3.713	0.686 0.192–2.446	4.270 * **1.881–9.691**	3.785 * **1.671–8.574**	1.208 0.448–3.258	1.011 0.442–2.312
**Sinusitis**	0.248 * 0.118–0.520	0.254 * 0.118–0.548	0.744 0.299–1.852	0.567 0.192–1.677	2.930 * **1.534–5.596**	2.304 * **1.207–4.399**	0.939 0.404–2.184	0.469 0.242–0.912
**Respiratory infection**	0.213 * 0.145–0.314	0.122 * 0.079–0.190	0.380 * 0.226–0.639	0.043 * 0.010–0.177	0.098 * 0.062–0.153	0.096 * 0.063–0.146	0.200 * 0.109–0.364	0.216 * 0.148–0.314

## Data Availability

The data presented in this study are available on request from the corresponding author due to privacy reasons.
